# The 1980s: d-AP5, LTP and a Decade of NMDA Receptor Discoveries

**DOI:** 10.1007/s11064-018-2640-6

**Published:** 2018-10-04

**Authors:** D. Lodge, J. C. Watkins, Z. A. Bortolotto, D. E. Jane, A. Volianskis

**Affiliations:** 1 Centre for Synaptic Plasticity, School of Physiology, Pharmacology and Neuroscience, University of Bristol, Bristol, UK; 2 School of Clinical Sciences, University of Bristol, Bristol, UK; 3 Centre for Neuroscience and Trauma, Blizard Institute, Barts and The London School of Medicine and Dentistry, Queen Mary University of London, London, UK

**Keywords:** Long-term potentiation (LTP), NMDA, NMDA receptors, APV, d-AP5, Synaptic plasticity

## Abstract

In the 1960s and 70s, biochemical and pharmacological evidence was pointing toward glutamate as a synaptic transmitter at a number of distinct receptor classes, known as NMDA and non-NMDA receptors. The field, however, lacked a potent and highly selective antagonist to block these putative postsynaptic receptors. So, the discoveries in the early 1980s of d-AP5 as a selective NMDA receptor antagonist and of its ability to block synaptic events and plasticity were a major breakthrough leading to an explosion of knowledge about this receptor subtype. During the next 10 years, the role of NMDA receptors was established in synaptic transmission, long-term potentiation, learning and memory, epilepsy, pain, among others. Hints at pharmacological heterogeneity among NMDA receptors were followed by the cloning of separate subunits. The purpose of this review is to recognize the important contributions made in the 1980s by Graham L. Collingridge and other key scientists to the advances in our understanding of the functions of NMDA receptors throughout the central nervous system.

## Introduction

**Fig. 1:**
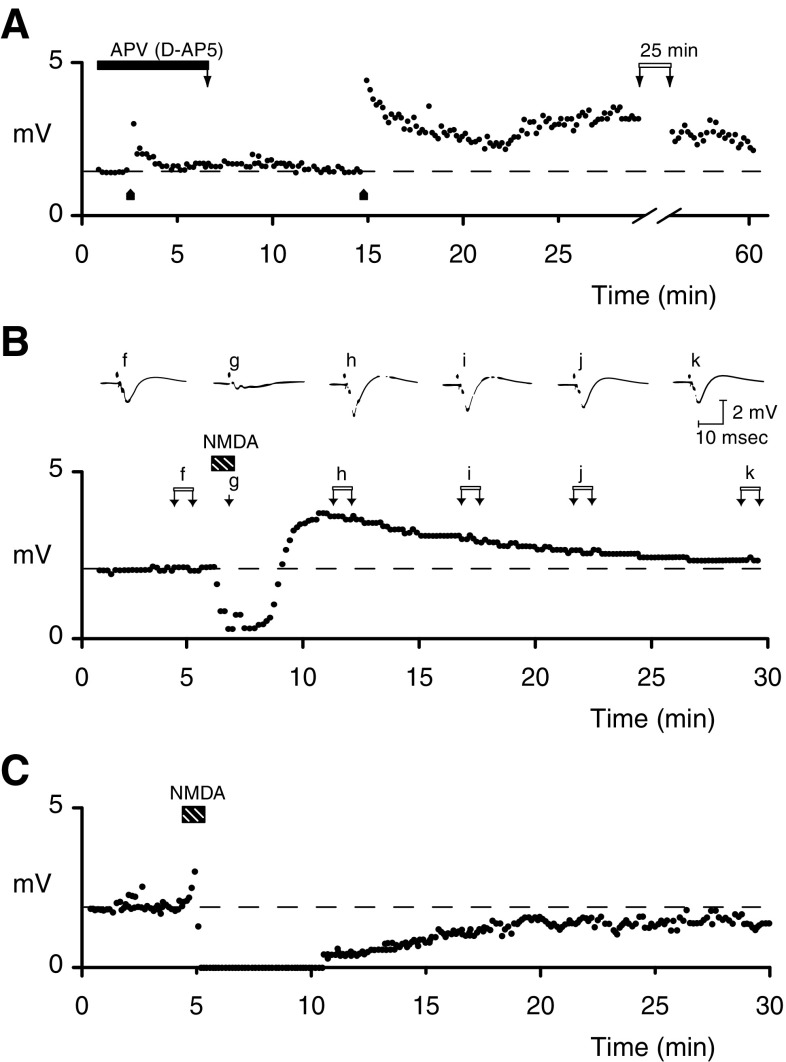
d-AP5, NMDA and NMDA receptor-dependent synaptic plasticity in 1983. **a** Iontophoretic application of d-AP5 blocks induction of LTP, which can be readily induced after washout of the antagonist [1]. **b** Brief iontophoretic application of NMDA leads to a transient enhancement of field potential amplitude, which declines to baseline over time [1]. **c** Longer, bath application, of NMDA leads to a permanent depression of synaptic transmission [49]

The 1980s proved to be a decade where *N*-methyl-d-aspartate (NMDA) receptor-mediated neurotransmission became firmly established. Perhaps this is epitomized by the 1983 paper published by Graham Collingridge and colleagues in the Journal of Physiology that changed our understanding of neuronal plasticity and, more widely, of the functional role of NMDA receptors in the central nervous system (CNS) [1]. With Steven Kehl and Hugh McLennan, they showed that a new selective NMDA receptor antagonist, 2-amino-5-phosphonovalerate (APV), inhibited the induction of long-term potentiation (LTP) of the synaptic input to CA1 neurones in hippocampal slices (Fig. 1a). Although cautious at the time, stating that ‘NMA receptors…may play a role in synaptic plasticity’, this observation, and the use of this new pharmacological tool are at the core of the now established role of NMDA receptors in excitatory neurotransmission, in many forms of synaptic plasticity and hence in learning and memory. In this brief review, we will consider what led up to this important discovery, what other related events surrounded it, and what directly followed from these studies with APV in the 1980s.

APV is now more commonly known as 2-amino-5-phosphonopentanoate (AP5). Although some studies state that either the racemic mixture, d,l-AP5, or the single active isomer, d-AP5, was used, it is unclear from some reports, however, which chemical entity was used. Thus, for simplicity and because d-AP5 is the active moiety within the racemate, d-AP5 has been used throughout the main body of this review.

## Background to 1980 Discoveries

In 1949, Hebb had proposed that changes in synaptic strengthening underlying learning required coincident pre- and post-synaptic activity [2] and, by the end of the 1960s, short lasting forms of synaptic plasticity were described in invertebrates and in the spinal cord. In their seminal review of 1968, Kandel and Spencer stated that ‘In contrast to the extensive data on spinal synapses, data on cortical synaptic plasticity are meager and, specifically, post-tetanic potentiation (PTP) has not yet been studied in detail comparable to that in the cord. This is unfortunate, since the complex morphology of cortical synapses may indicate a capability for unusual plastic alteration.’ Indeed, although long lasting depression [3] and facilitation [4] were already observed in hippocampal synapses there were very few other accounts of synaptic plasticity in the mammalian brain [5]. The phenomenon of long-term potentiation (LTP) was first detailed by Bliss and Lomo [6] in the dentate gyrus in vivo. Soon, however, hippocampal slices [7] became the preferred preparation for studying LTP [8–10]. LTP was shown to require cooperativity between strong afferent input from many fibres and a resulting strong depolarization of the postsynaptic neurone [11, 12]. Such potentiation was input specific so that other afferent inputs were unaffected [9] or reduced, i.e. heterosynaptic depression [13]. By contrast, a low rate of stimulation could lead to a long-term depression of all inputs [12]. The nature of the chemical transmitters involved in such processes was largely conjectural.

In the late 1970s, the concept emerged of different subtypes of glutamate receptor that mediate synaptic excitation in the central nervous system [14, 15]. Initial observations with several natural and recently synthesized acidic amino acids indicated that that *N*-methyl-d-aspartate (NMDA) was a considerably more potent excitant of central neurones than l-glutamate and l-aspartate [16, 17]. An early indication that there might be subtypes of receptors for these acidic acids was the finding that the ratios of potency between d,l-homocysteate or l-aspartate and l-glutamate, and later between NMDA and kainate, varied between different neuronal populations [18–20]. These findings were part of the developing concept of subtypes of glutamate receptors. Studies with other structurally constrained glutamate analogues from natural resources, such as kainic, domoic and quisqualic acids, suggested potential diversity of receptors mediating synaptic excitation. This diversity was supported by the observation that Mg^2+^ reduced the effectiveness of NMDA to a greater extent than most other glutamate analogues [21]. Further development of this concept required discovery of suitable antagonists. Longer chain analogues of glutamate, namely α-amino-adipic and -suberic and diaminopimelic acids, were weak, selective antagonists of NMDA-induced excitation rather than that induced by quisqualate, kainate and α-amino-3-hydroxy-5-methyl-4-isoxazolepropionic acid (AMPA) and reduced some synaptic events [22–27]. Thus the concept of NMDA and non-NMDA (later to be known as AMPA and kainate) receptors became accepted [28].

By the mid-1970 s, there were a number of papers reporting the presence, uptake and calcium-dependent release of l-glutamate and/or l-aspartate, which provided powerful evidence toward a transmitter role for these acidic amino acids [29] but the lack of selective, potent and established receptor antagonists slowed further progress in this field.

### d-AP5, NMDA and LTP

Hence, the description of 2-amino-phosphonovaleric acid [30] as a potent and selective NMDA receptor antagonist at synapses on spinal neurones was the breakthrough needed to allow a thorough investigation of the physiological role of NMDA receptors.

Collingridge, being a Bristol graduate with Jeff Watkins, a PhD student with John Davies and a postdoc with Hugh McLennan, was in a good position to examine the effects of this new pharmacological tool on hippocampal slices in vitro, a preparation being used for detailed electrophysiology. Thus, Collingridge and collaborators first demonstrated that d-AP5, a gift from Jeff Watkins, was a more potent and selective NMDA receptor antagonist than previously used compounds, the activity lying mainly in the d-isomer [31]. With his co-authors, he then went on to show that d-AP5 reduced the synaptic potentiation in the CA1 region that followed high frequency stimulation of the Schaffer collateral input (Fig. 1a) with minimal effect on synaptic potentials at low stimulation frequencies [1]. Thus, the role of NMDA receptors in the initiation of LTP following high frequency stimulation was established in this highly quoted paper (1830 citations; Web of Science; September 2018).

This basic observation, in hippocampal slices, of the role of NMDA receptors in synaptic plasticity was rapidly seized upon and replicated by other major researchers in LTP using different paradigms but with a common d-AP5-sensitive theme: Schaffer collateral/commissural pathways to CA1 [32–35], perforant pathway to dentate gyrus in vivo [36, 37]. However, LTP at some hippocampal synapses appeared not to be mediated by NMDA receptors. For example, only the commissural, and not the mossy fibre, input to CA3 was sensitive to d-AP5 [38].

The use of d-AP5 allowed the role of NMDA receptors in LTP to be extended to rat visual cortical slices, although in this tissue GABAergic inhibition appears to play a more important modulating role than in the hippocampus [39]. In parallel, Wolf Singer’s group showed that, at a critical period of development in the kitten visual cortex, d-AP5 also prevented the normal developmental process of activity-dependent modifications, which results in orientation selectivity of neurones in the visual cortex [40, 41]. NMDA receptor antagonists prevent both the loss of inappropriate synaptic connections and the strengthening of correct connections. Another form of learning during development mediated by NMDA receptors is imprinting in day-old chicks, a phenomenon in which both the learning itself and the subsequent increase in glutamate binding are sensitive to d-AP5 [42, 43].

Concurrent with these observations in mammals, the development of a retinotopic map in the tectum of frogs and goldfish was also reported to be impaired by d-AP5 [44–46]. Part of this re-wiring may require the growth of neurites and dendrites as well as cell survival processes that are also NMDA receptor-dependent [47, 48]. Interestingly both the ability to induce cortical LTP and the density of NMDA receptors appeared to peak during this critical period for development of cortical connections, stressing the importance of NMDA receptors in this form of plasticity [49].

### Is NMDA Receptor Activation Sufficient for Inducing LTP?

The discovery that d-AP5 blocked induction of LTP suggested that application of NMDA alone should be sufficient to induce plasticity. As shown in the original paper, brief exposure to NMDA results only in a transient enhancement of field potentials (Fig. 1b, [1]). In contrast, a longer application of NMDA (Fig. 1c, [50]) or glutamate [50] resulted in a depression of synaptic transmission, later recognized as NMDA receptor-dependent chemical LTD [51]. Similarly, low frequency afferent stimulation, besides limiting the induction of LTP [52], can also induce a long-term depression of synaptic transmission [12], shown in the 1990s to be d-AP5-sensitive [53, 54].

The transient enhancement of the amplitude of the field potentials, seen following NMDA application (Fig. 1b), seemed similar to the initial decremental phase of LTP (Fig. 1a), termed short-term potentiation (STP); STP, just like LTP, was d-AP5-sensitive raising the question whether STP was essential to the establishment of LTP or whether it was a mechanistically distinct parallel event [1, 55, 56]. Eventually it was shown that NMDA receptors of different subunit composition mediate induction of STP versus LTP [57] and that NMDA-induced enhancement of the field potential amplitude is distinct from STP, which is associated with a change in slope of field responses [58]. Gary Lynch’s group, did, however, show that successful induction of chemical LTP could be achieved when application of NMDA was followed by a brief application of d-AP5 [59], the antagonist possibly preventing the longer activation of NMDA receptors required for the induction of LTD, thus revealing the chemical LTP.

### Why NMDA Receptors for LTP?

Understanding why NMDA receptors play a unique role in synaptic potentiation depended on two key observations.

The first relates to the explanation of (i) the strange current–voltage curve of the NMDA receptor [60] and (ii) why Mg^2+^ ions inhibit responses to bath application of NMDA [21]. The discovery was that Mg^2+^ ions produce a voltage-dependent brake on channel conductance particularly at hyperpolarised membrane potentials [61, 62]. The Schaffer collaterals release glutamate onto both NMDA and AMPA receptors, the latter dominating the synaptic potential because of the Mg^2+^ block of the NMDA receptor. Removal of Mg^2+^ ions uncovered a slow NMDA component of the EPSP [63]. The NMDA receptor component also rises more slowly than the AMPA receptor component, which decays quickly not giving sufficient time for the Mg^2+^ block to be fully removed. The depolarisation resulting from AMPA receptor activation is not an absolute requirement: with AMPA receptors blocked, a slow synaptic depolarisation mediated by NMDA receptors is uncovered [64–67]. Thus the depolarization that follows temporal (or spatial) summation during high frequency stimulation of excitatory synaptic inputs is required to relieve the Mg^2+^ ion block, which immediately increases the conductance of the NMDA receptor-coupled channel [68, 69]. This slow NMDA receptor component can be observed during high frequency stimulation beneath the AMPA receptor-mediated synaptic potentials [70].

The second key observation is that NMDA receptors are readily permeable to calcium when the voltage-dependent Mg^2+^ ion block is relieved [71]. The resultant increase in intracellular calcium, which can be visualised in dendritic spines receiving NMDA receptor activation [72] is the main driving force for plasticity in LTP induction protocols [73, 74]. Calcium activates a complex array of secondary intracellular events, including up-regulation of AMPA receptors at the potentiated synapse [75–78] and activation of protein kinases [79–82], that act as molecular switches [83, 84] and that also regulate protein synthesis dependence of the late phases of LTP [85, 86]. Much of this, including recruitment of glutamate receptors to dendritic spines, was debated early [87, 88] and detailed in subsequent decades [89–91]. Nevertheless, although the field was in general agreement about the central role of NMDA receptors in initiating LTP, there was little consensus about the mechanisms of LTP expression, which could be mediated by pre-synaptic and post-synaptic mechanisms alike [92]. The differences in the outcome of various NMDA receptor activation protocols depends among others on the extent to which different intracellular messaging systems are engaged and the type of synaptic plasticity that is induced or maintained [93, 94].

A further factor to consider is the role of inhibitory synapses, which are recruited when afferent pathways are stimulated with a tetanic pattern, including Schaffer collateral-commissural fibres into CA1. GABAergic hyperpolarisation helps maintain the Mg^2+^ brake on the NMDA receptor conductance. Blocking GABA-A receptor-mediated inhibition reveals the NMDA receptor component at low and high frequencies of stimulation [68, 95] and facilitates LTP [96, 97]. During high frequency bursts, postsynaptic GABAergic inhibition declines and hence allows calcium flux through NMDA receptor channels [98]. The more natural theta stimulation allows very short trains of stimuli to induce LTP [99, 100] in which postsynaptic GABAergic inhibition is less prominent, itself being regulated by presynaptic GABA-B receptor-mediated inhibition [93, 94].

Temporal summation or frequency dependence of the recruitment of NMDA receptors, the resultant dendritic depolarization and calcium entry are the driving forces of LTP [70, 101]. Thus, NMDA receptors function as coincidence detectors that sense synchronised pre- and post-synaptic activity and uniquely allow for the Hebbian principle of cooperativity, between strong afferent input and marked postsynaptic depolarization, which is required for synaptic strengthening [68, 69, 102, 103]. This aspect of cooperativity can be side-stepped, as described above, by a small postsynaptic depolarization, reducing extracellular Mg^2+^ ion concentration or reducing post- or pre-synaptic GABAergic inhibition, when low frequency stimulation can induce LTP [68, 103–105].

### Ubiquity of NMDA Receptors

As is apparent from the above sections, NMDA receptors are not unique to the Schaffer collateral synapse on the CA1 hippocampal pyramidal neurones. The development of d-AP5, as a potent and selective NMDA receptor antagonist, allowed the role of NMDA receptors to be more widely investigated.

Indeed reports of a transmitter role for NMDA receptors onto spinal neurones in vivo using weaker NMDA receptor antagonists (see above) preceded the hippocampal papers. Interestingly using d-AP5, a single stimulus of peripheral afferents, unlike the initial reports in the hippocampus [1], could evoke NMDA receptor-mediated synaptic potentials in spinal neurones [30, 106]. The causal features of this difference are likely to be the more depolarized state in vivo and the temporal and spatial summation that occurs following stimulation of a mixed population of primary afferents and internuncial neurones in the spinal cord experiments. Frequency-dependent depolarization and potentiation, sensitive to NMDA receptor antagonists such as d-AP5, are also seen in these spinal pathways [107, 108].

Throughout the 1980s, d-AP5 was used to demonstrate a transmitter role for NMDA receptors throughout the brain; substantia nigra [109], dentate gyrus [110], interpeduncular nucleus [111], cerebellar Purkinje cells [112], neocortical neurones [113], red nucleus [114] and ventro-basal thalamus [115] as well as in sympathetic ganglia [116].

Most of the above studies were performed on rats or other mammals, although NMDA receptor-mediated d-AP5-sensitive synaptic excitation was earlier demonstrated in the spinal cord of amphibians [117–119] and of fish [120, 121] and in the retina of fish [122, 123]. Interestingly, superfusion of the exposed spinal cord with NMDA is able to initiate fictive locomotion in both frogs and lampreys, a pattern blocked by d-AP5 [118, 120].

Thus it became apparent in the 1980s that NMDA receptors were important mediators of synaptic transmission throughout the central nervous system of vertebrates, although the question of the natural transmitter was still unanswered. l-Aspartate and l-glutamate, although mimicking the effects of NMDA, were considerably less potent than NMDA itself in most assays, despite biochemical evidence supporting a transmitter role for these two amino acids [29]. Two key observations in the 1980s supported l-glutamate as the candidate. Firstly, in the absence of amino acid transport processes, l-glutamate became tenfold more potent than NMDA on dissociated neurones [124]. Secondly, in binding studies, l-glutamate was tenfold more potent than l-aspartate as an inhibitor of radioactive d-AP5 binding to NMDA receptors in rat brain membranes [125] and had an indistinguishable autoradiographic distribution to d-AP5 in rat brain [126].

### Epilepsy

Epilepsy results from changes in brain circuitry excitability that lead to bursts of cortical activity arising spontaneously or from otherwise subthreshold events. A prime example of such epileptogenesis is the phenomenon of kindling, a form of plasticity following repetitive brain stimulation that leads to epilepsy-like convulsions. Kindling has features in common with LTP [127, 128]. In particular, d-AP5 prevents the induction of the epileptiform activity, but also reduces the resultant seizure-like discharges, following kindling protocols [129, 130].

However, the first real evidence of the role of NMDA receptors in epilepsy came from in vivo studies in Harry Bradford’s and Brian Meldrum’s laboratories. They showed that local administration of d-AP5 reduced seizures resulting from a cobalt-induced lesion [131] and both sound-induced seizures in DBA-2 mice and pentylenetetrazol-induced seizures in Swiss mice [132] as well as photic stimulated epilepsy in primates [133]. The striking correlation in potency between NMDA receptor antagonism in vitro and that against seizures of three competitive NMDA receptor antagonists substantiated the importance of NMDA receptors as anticonvulsants [132].

This was followed by many publications showing that both induction and maintenance of many forms of epileptiform activity in hippocampal slices [95, 134–137] and in cortical slices [138, 139] could be prevented by d-AP5. This included the blocking of ex vivo bursting epileptogenic foci in kainate-lesioned rat hippocampi [140] and in surgically removed human neocortex [141]. The bursting pattern of layer 4/5 neurones during slow wave sleep was also blocked by local ejection of d-AP5 [142].

The role of NMDA receptors in, and the use of NMDA receptor antagonists for, various forms of epilepsy is still a subject of therapeutic interest.

### Pain

Another therapeutically important aspect of plasticity is the development of neuropathic and other chronic pain conditions, for example phantom limb pain. Such maladapted plasticity may lead to hyperalgesia and allodynia, two symptoms that indicate nociceptive pathways have been abnormally strengthened or new ones formed.

Because, as mentioned earlier, the polysynaptic excitation of spinal neurones following afferent stimulation of hind limb nerves is mediated by NMDA receptors [22–24, 30, 106], it was not surprising that NMDA and d-AP5, respectively, induced and reduced nociceptive responses following local application to the spinal cord [143–145].

Concerning plasticity, the phenomenon of ‘wind-up’ whereby repetitive nociceptor fibre stimulation leads to a potentiated response of spinal neurones [146] is thought to underlie central sensitization leading to hyperalgesia. This form of plasticity is prevented by d-AP5 following local spinal application in vivo [107] and following bath application in vitro [147]. Unilateral foot paw tissue damage may result in secondary hyperalgesia in the contralateral limb, which can be prevented by spinal administration of d-AP5 [148]. Nevertheless weak bioavailability of d-AP5 has limited its use in vivo for researching the role of NMDA receptors in various pain states.

### d-AP5 and Memory

Because LTP is thought to be one of the mechanisms underlying learning and memory, it was not surprising that the effects of d-AP5 were assessed in learning paradigms rapidly following the description of its block of LTP [1]. Parallels had already been drawn between the rate of decline of LTP and of loss of memory in older rats (reviewed in [149]).

Because of the low bioavailability of competitive NMDA receptor antagonists in general, d-AP5 was injected into the cerebral ventricles (i.c.v.) in the early experiments of Richard Morris and collaborators. Treated and untreated rats, placed in a large pool of opaque water, were compared for their ability to learn the position of a hidden escape platform over a 5 day period, a task now known as the Morris Water Maze. The results were highly significant in that the d-AP5 treated animals took much longer to learn the location of the platform, spending much less time than the controls in the correct quadrant of the pool [36]. d-AP5-treated animals did not show deficits in a visual discrimination test suggesting a role for NMDA receptors specifically in spatial learning, which is thought to be a hippocampus-based phenomenon. By changing the time of administration of d-AP5, they were able to show that, in parallel with LTP, NMDA receptors were required for the acquisition or encoding of memory but not for its storage or retrieval [150], see also [151].

Similarly, i.c.v. d-AP5 disrupted acquisition of short term memory (radial maze) and attenuated retention of long term memory (passive avoidance) provided the drug was injected before the learning phase [152] and prior administration of d-AP5 could result in memory decline in an operant learning paradigm [153]. Acquisition of odour discrimination was also prevented by d-AP5 but previously learned memories were not disrupted [154]. These data extended the concept of an NMDA receptor-mediated LTP-like plasticity requirement from the hippocampal-based spatial domain to other forms of learning and memory.

### Neurotoxicity and Neuroprotection

In contrast to its positive role in neuroplasticity, excess NMDA receptor activation can lead to d-AP5-sensitive neurodegeneration [155, 156]. This sensitivity to NMDA-induced neurotoxicity varies between populations of neurones, a finding likely related to the differences in NMDA receptor expression and/or calcium buffering [157]. Release of glutamate following excessive and/or prolonged stimulation of neuronal pathways can also result in d-AP5-sensitive degeneration of targetted neurones [158] similar to that following epileptiform activity in hippocampal slices [159, 160].

Brain ischaemia and hypoglycaemia lead to high extracellular levels of glutamate [161–163]. Although competitive NMDA receptor antagonists have been shown to be effective in reducing neuronal cell loss following temporary carotid artery occlusion [164] and hypoglycaemia [165], the hope for them as clinical agents [166] has not yet been realized.

### Beyond d-AP5: Medicinal Chemistry Around the NMDA Receptor

Although the synthesis of NMDA was first reported in 1962 [167], it wasn’t until much later that chemists developed more potent agonists by conformational restriction of either aspartate or glutamate (reviewed in [174]). Such agonists include α-tetrazolylglycine [168], the cyclobutane *trans*-ACBD [169, 170] and the cyclopropanes d-CCG-II and l-CCG-IV [171–173].

Following the success of d-AP5 in forwarding our understanding of the role of NMDA receptors, medicinal chemists in academia and industry continued to develop new compounds in order to increase potency and/or bioavailability (reviewed in [174]). Increasing the affinity of d-AP5 was achieved by conformational restriction for example by incorporating a double bond into the side chain (e.g. CGP 37849 and its α-carboxyethyl ester CGP 39551, [175]), or incorporating the α-amino group and some of the side chain into a piperidine ring (e.g. CGS 19755, [176]). Like d-AP5, D-AP7, a longer chain analogue, was also found to be a competitive NMDA receptor antagonist and blocked LTP whereas d-AP4, D-AP6 and D-AP8 were essentially inactive [32, 177, 178]. Conformational restriction of D-AP7 led to the development of high affinity antagonists such as the piperazine derivatives d-CPP [179, 180] and d-CPPene [181], the decahydroisoquinoline LY274614 [182] and the phenylalanine SDZ EAB515 [183].

Several of these high affinity NMDA receptor antagonists were radiolabelled (e.g. [^3^H]AP5 [125], [^3^H]CPP [184], [^3^H]CGS19755 [185] and [^3^H]CGP 39653 [186]). They were used in binding assays and alongside [^3^H]glutamate [187, 188] and [^3^H]MK-801 [189] (a high affinity channel blocker) in autoradiography, to study the distribution of native NMDA receptors throughout different brain regions.

High affinity NMDA receptor antagonists were used in animal models of CNS disorders and were found to be anticonvulsant in models of epilepsy, neuroprotective in models of cerebral ischaemia and to be effective in models of chronic pain. Some, such as d-CPPene, also were taken into clinical trials for prevention of brain damage following stroke or head injury and for treatment resistant forms of epilepsy. Positive outcomes from such clinical trials, e.g. with d-CPPene and CGS19755, have been prevented by the occurrence of side effects, particularly of a psychogenic nature [190, 191] .

### Coincidental but Related Pharmacological Discoveries of the 1980s

Interestingly, Collingridge and collaborators were not the only group studying LTP pharmacologically in 1983. Patrice Guyenet’s laboratory was independently showing that the effects of phencyclidine, ketamine and sigma opiates blocked the long term potentiation of the population spike in CA1 region of the hippocampal slices [192, 193]. Equally independent was the observation that phencyclidine, ketamine and sigma opiates were selective NMDA receptor antagonists on spinal neurones in vivo [194–196]. Thus, these two independent groups coincidentally provided extra support for the role of NMDA receptors in LTP [1].

Unlike competitive NMDA receptor antagonists, ketamine blocks within the receptor-coupled channel [197], pharmacologically mimicking the voltage-dependent block of Mg^2+^ ions but with slower kinetics. Although there are concerns related to the specificity of ketamine and phencyclidine as NMDA receptor antagonists, particularly at higher concentrations [198], its rapid CNS bioavailability and reversibility following systemic administration makes low doses of ketamine particularly useful for studying the effects of NMDA receptors in vivo.

By the 1980s, many of the pharmacological and clinical properties of ketamine were already established in the absence of knowledge of it as an NMDA receptor antagonist. Developed as an anaesthetic, it was known for its good analgesia and its safety but with recognized emergence phenomena including hallucinations [199]. In the 1980s striking similarities between the actions of ketamine and d-AP5 emerged. For example, ketamine’s effect on polysynaptic responses of spinal neurones [194, 195], on hippocampal LTP [192], on spinal ‘wind-up’ [108], on cortical synaptic transmission [200], on cortical epileptiform activity [201], on sound-induced seizures [202] and on ocular dominance in the visual cortex [203] echoed the effects of d-AP5 cited above.

Another major aspect of the NMDA receptor’s pharmacology was discovered in the 1980s. Philippe Ascher’s group showed that glycine, or a glycine-like substance such as d-serine, was a required co-agonist for NMDA receptor activation [204]. This glycine-site was not sensitive to the traditional inhibitory antagonist, strychnine. Instead, compounds such as HA-966 and 7-chlorokynurenate were shown to be NMDA receptor antagonists acting via this glycine site (for example [205–208]).

It is beyond the scope of this review to describe all the contributions that using ketamine and other non-competitive NMDA antagonists including glycine-site antagonists have made to our understanding of the importance of NMDA receptors. Some of this literature, concerning the effects of ketamine in synaptic plasticity, neuroprotection, epilepsy, pain and behaviour, is cited in previous reviews [198, 209].

### Heterogeneity Within NMDA Receptors

Also in the 1980s, the possibility of subtypes of the NMDA receptor was first raised. Differential sensitivity of brain regions to quinolinic acid, a weak naturally occurring NMDA receptor agonist [210–212] suggested NMDA_1_ and NMDA_2_ receptor subtypes. Similarly, regional differences in the sensitivity to glycine and to a variety of NMDA receptor antagonists [213] and to differential stimulation of [^3^H]MK-801 binding by l-glutamate in different brain regions [214, 215] and relative affinity of various competitive antagonists in autoradiography studies [216, 217] reinforced the idea of heterogeneity in NMDA receptor subtypes. Specific profiles were noted between the rat medial thalamus, the forebrain and the cerebellum [218].

Such suggestions pre-dated the cloning of NMDA receptor subunits in the early 1990s, which confirmed this heterogeneity. The first cloned subunit [219] is now called GluN1 and is the glycine-sensitive subunit. Cloning of the four glutamate-sensitive subunits, GluN2A-D followed soon [220] and of two more glycine-sensitive GluN3 subunits followed later (reviewed in [221]). Defining the roles of the NMDA receptor subunits in aspects of plasticity has become a major interest of Collingridge and many others [57, 222–230].

## Conclusions

**Fig. 2:**
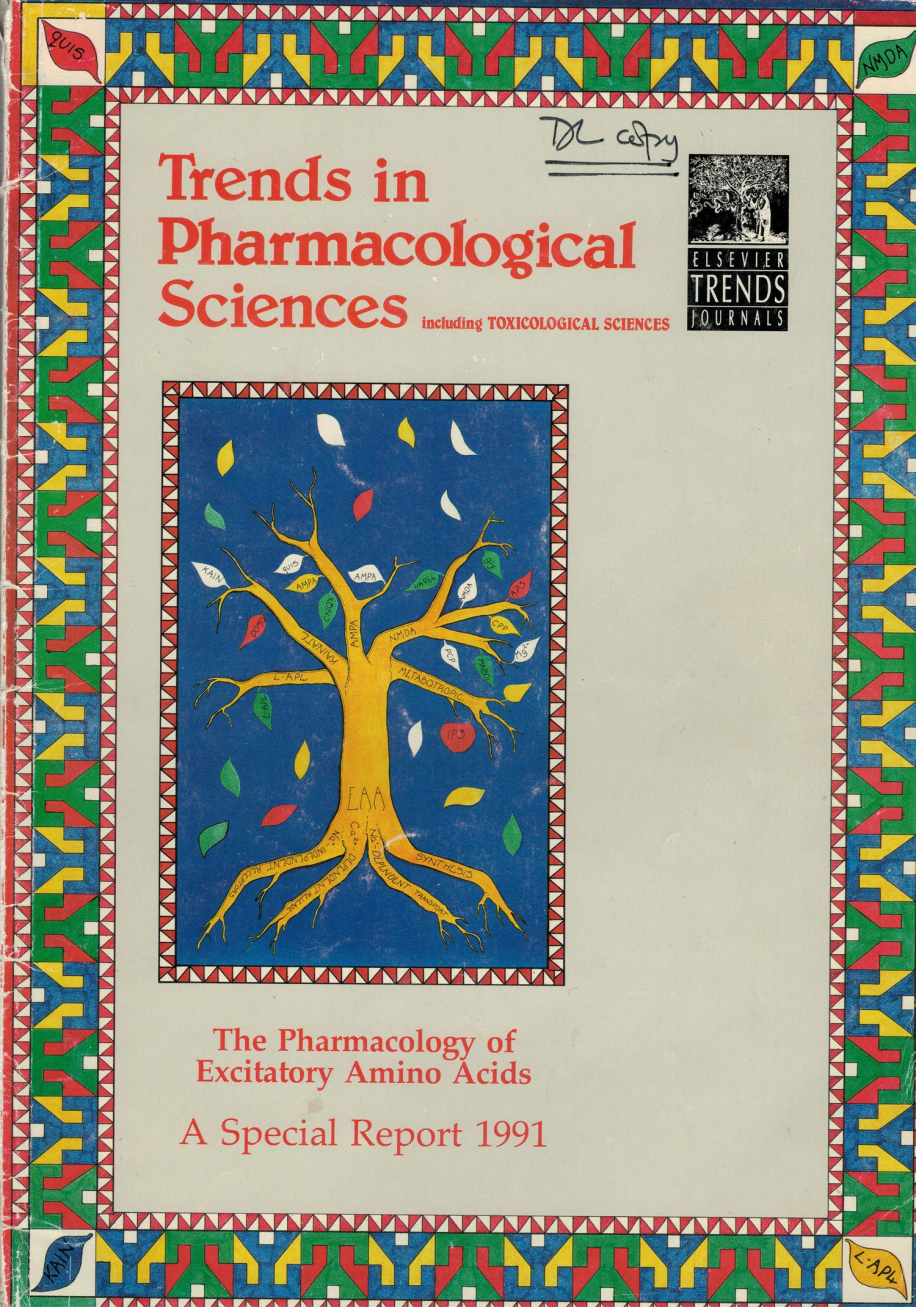
Trends in pharmacological sciences: Special Report 1991. Cover page: This supplement was a compilation of the articles published each month during 1990 on the theme: “The Pharmacology of Excitatory Amino Acids” edited by David Lodge, and Graham L. Collingridge with Alison Abbott of Elsevier. The supplement was sponsored by Leslie L. Iversen of Merck & Sharp and Dohme Research Laboratories. The Glutamate Tree of Life is represented in the “Cover design by Nigel Hynes, based on an original idea of David Lodge”

**Fig. 3:**
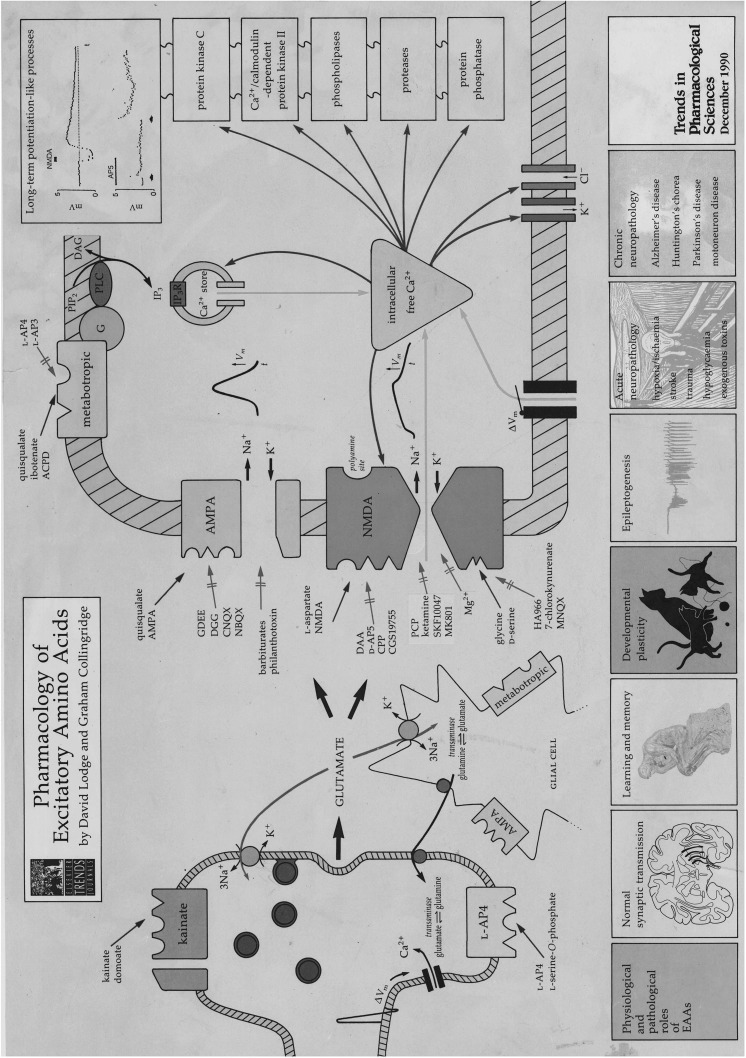
Pharmacology of excitatory amino acids: 1991 Poster. This accompanying Poster summarised what had been published in the Special Report. Much of what was simplistically sketched in 1991 still holds true today in 2018 with major advances that have been made in many areas. In particular our knowledge about glutamate receptor involvement in disease has shown huge advances together with advances in the molecular biology, crystal structure, genetics and intracellular signalling of glutamate receptors. Absence of metabotropic glutamate receptors on the illustrated glutamatergic terminal and of pharmacological tools for these G-protein coupled receptors are obvious omissions. Two areas of neuropsychiatry, namely schizophrenia and depression, are now widely linked with NMDA receptor function

The growing evidence of the role of glutamate and of NMDA receptors in particular, in synaptic transmission received a considerable boost in the 1980s. This was largely driven by the discovery of the highly selective NMDA receptor antagonist, d-AP5, which enabled its use to establish a role for NMDA receptors in synaptic transmission and plasticity [1, 30]. This review has focussed on some examples of the resulting explosion in knowledge, which were more thoroughly described in a 1991 Supplement of Trends in Pharmacological Sciences, which also included a poster depicting pharmacological tools that were available for targeting of glutamate receptors, synaptic transmission and plasticity Figs. 2, 3.
